# Effects of Cannabidiol on Locomotor Activity

**DOI:** 10.3390/life12050652

**Published:** 2022-04-27

**Authors:** Fabrizio Calapai, Luigi Cardia, Gioacchino Calapai, Debora Di Mauro, Fabio Trimarchi, Ilaria Ammendolia, Carmen Mannucci

**Affiliations:** 1 Department of Chemical, Biological, Pharmaceutical and Environmental Sciences, University of Messina, 98122 Messina, Italy; fabrizio.calapai@unime.it; 2 Department of Human Pathology of Adult and Childhood “Gaetano Barresi”, University of Messina, Via C. Valeria, 98125 Messina, Italy; luigi.cardia@unime.it; 3 Department of Biomedical and Dental Sciences and Morphological and Functional Imaging, University of Messina, 98125 Messina, Italy; ddimauro@unime.it (D.D.M.); fabio.trimarchi@unime.it (F.T.); ilaria.ammendolia@unime.it (I.A.); cmannucci@unime.it (C.M.)

**Keywords:** cannabidiol, locomotor activity, motor activity, locomotion, cannabis

## Abstract

Cannabidiol (CBD) is the second cannabinoid, in order of importance after Δ9-tetrahydrocannabinol (THC), from *Cannabis sativa*. Unlike THC, CBD does not cause psychotomimetic effects, and although these compounds have the same chemical formula, their pharmacological characteristics are not equivalent. Preclinical studies suggest that CBD has anti-inflammatory, analgesic, anxiolytic, antiemetic, anticonvulsant, and antipsychotic properties and influences the sleep–wake cycle. The evaluation of effects on spontaneous motor activity is crucial in experimental pharmacology, and the careful measurement of laboratory animal movement is an established method to recognize the effects of stimulant and depressant drugs. The potential influence of CBD on locomotor activity has been investigated through numerous in vivo experiments. However, there is no clear picture of the impact of CBD on these issues, even though it is administered alone for medical uses and sold with THC as a drug for pain caused by muscle spasms in multiple sclerosis, and it was recently licensed as a drug for severe forms of infantile epilepsy. On this basis, with the aim of developing deeper knowledge of this issue, scientific data on CBD’s influence on locomotor activity are discussed here. We conducted research using PubMed, Scopus, Google Scholar, and a search engine for literature between January 2009 and December 2021 on life sciences and biomedical topics using the keywords “motor activity”, “locomotor activity”, and “locomotion” in combination with “cannabidiol”. In this article, we discuss findings describing the effects on locomotor activity of the CBD precursor cannabidiolic acid and of CBD alone or in combination with THC, together with the effects of CBD on locomotor modifications induced by diseases and on locomotor changes induced by other substances.

## 1. Introduction

Cannabidiol (or 2-[(6R)-6-Isopropenyl- 3-methyl-2-cyclohexen-1-yl]-5-pentyl-1,3-benzene-di-ol) (CBD) is a chemical compound that was isolated by Adams in 1940 from *Cannabis sativa* [1]; its structure was elucidated in 1963 by Mechoulam [2] et al.

**Figure 1:**
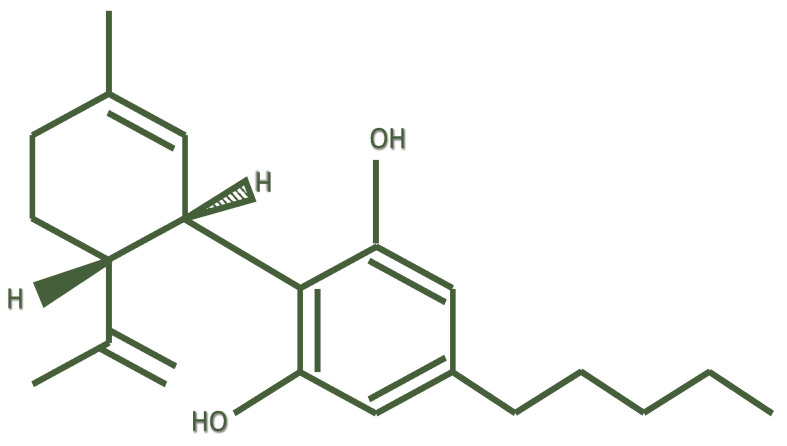
Chemical structure of cannabidiol.

Following Δ9-tetrahydrocannabinol (THC), cannabidiol is the second most important phytocannabinoid, having relevant pharmacological activity [3]. CBD is structurally different from its precursor cannabidiolic acid (CBDA) by a carboxyl group (–COOH). It is the result of decarboxylation of CBDA under the effects of heat [4]. CBD is a meroterpenoid derived from the alkylation of an alkyl resorcinol with a monoterpene unit [5] (Figure 1). CBD and THC share the same chemical formula, with 21 carbon atoms, 30 hydrogen atoms, and two oxygen atoms. However, while THC exists in a planar conformation, CBD shows a different conformation in which the two rings are more or less at right angles to each other. Consequently, CBD does not bind or activate the CB1 receptor, thus explaining the lack of THC-like psychotomimetic effects [6].

CBD is a lipophilic molecule, is quickly absorbed, and easily crosses biological barriers [7]. CBD induces CYP1A2 and CYP2B6 enzymatic activity and inhibits that of CYP2C8, CYP2C9, CYP2C19, and of uridine-glucuronosyltransferases; consequently, when consumed concomitantly with other drugs, undesired interactions can occur [8]. 

CBD undergoes hepatic oxidative and conjugative metabolism by CYP and uridine 5′-diphospho-glucuronosyltransferase (UGT) enzymes, respectively [9]. The primary metabolic reactions of CBD are allylic hydroxylations at the 6- and 7-positions that are catalyzed by cytochrome P450 (CYP) [10]. CBD metabolism includes further oxidations with the formation of numerous metabolites. Among these, the major metabolite is 7-COOH-CBD, which, in turn, is oxidized at the side chain [11].

There is evidence that the presence of CBD in cannabis preparations can work as a regulator of the effects of THC, acting as an antagonistic allosteric modulator at the cannabinoid (CB)1 and CB2 receptors [12,13]. Other findings support the hypothesis that CBD modulates THC activity through mechanisms independent of CB1 receptor involvement [14]. CBD seems to be an indirect activator of the CB1 receptor through the increase in endogenous cannabinoid anandamide levels, and it has also been identified as a negative allosteric modulator of the same receptor [15]. Other experiments suggest that CBD could act as an inverse agonist or antagonist of the cannabinoid type 2 receptor (CB2R) [16]. Instead of THC, CBD does not cause psychotomimetic effects, and although these compounds have the same chemical formula, their pharmacological characteristics are not equivalent [17].

CBD is not a full ligand for CB1 or CB2 receptors, having low affinity for these targets [16]. CBD modulates other cellular pathways, such as the transient receptor potential subfamily V member 1 (TRPV1) cation channels [18], GPR55 receptor [19], the enzyme fatty acid amide hydrolase (FAAH) [20], serotonin 1A (5-HT1A) receptor [21], peroxisome proliferator-activated receptor (PPAR)-gamma [22], adenosine uptake [23], calcium channels [24], and opioid receptors [25]. 

**Figure 2:**
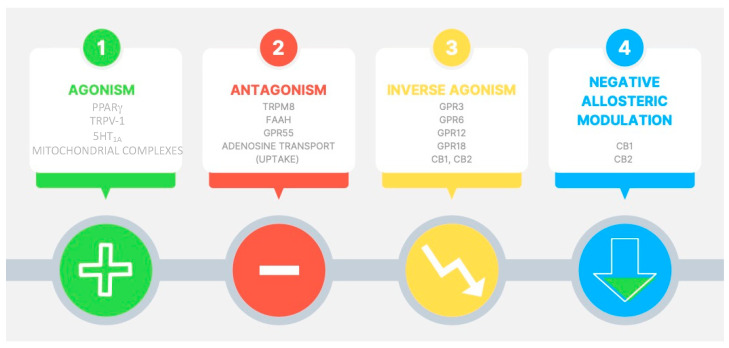
Pharmacological targets potentially involved in cannabidiol effects. 5-HT_1A_ = serotonin 1A receptor; PPAR-γ = peroxisome proliferator-activated receptor gamma; TRPV-1 = transient receptor potential cation channel subfamily V member 1; TRPM8 = transient receptor potential cation channel subfamily M (melastatin) member 8; FAAH = fatty acid amide hydrolase; GPR55 = G protein-coupled receptor 55; GPR3 = G protein-coupled receptor 3; GPR6 = G protein-coupled receptor 6; GPR12 = G protein-coupled receptor 12; GPR18 = G protein-coupled receptor 18; CB1 = cannabinoid receptor type 1; CB2 = cannabinoid receptor type 2.

Regarding the in vitro effects of cannabinoids, antioxidant activity has been associated with the content of the phenolic group in their structures, which occurs in CBD regardless of its activity as a ligand for CB1 [26], and the regulatory role of CBD in intracellular Ca^2+^ signaling has been demonstrated [27]. Moreover, it has been reported that CBD could protect dopaminergic neurons and cerebellar granule neurons from mitochondrial dysfunction induced by 1-methyl-4-phenylpyridinium and rotenone, respectively [28]. Multitarget mechanistic pathways of action of CBD are summarized in Figure 2. 

Preclinical studies suggest that CBD could be useful due to its anti-inflammatory, analgesic, anxiolytic, antiemetic, anticonvulsant, antipsychotic, and neuroprotective properties and its influence on the sleep–wake cycle [29,30,31]. CBD has been shown to have several pharmacological and biological properties suitable for medical use and a relatively safe profile [32]. 

Among these properties, the potential influence on locomotor activity has been investigated through various in vivo experiments. Interestingly, in 2018 the World Anti-Doping Agency (WADA) removed CBD from the list of prohibited substances for use outside of competition, leaving it to be freely use by athletes. This decision was taken without an in-depth analysis of the real effects of CBD on sport performance or locomotor activity. However, CBD is contained in many products sold as dietary supplements on internet sites, even though this compound is still not definitively listed as a food in this category by regulatory authorities in Europe and North America [33]. The evaluation of effects on spontaneous motor activity is crucial in experimental pharmacology, and the careful measurement of laboratory animal movement is an established method to recognize stimulant and depressant drug effects [34]. The influence of CBD on motor activity can be due to direct effects on motor function or through interaction with other drugs influencing movement or through potential effects in healthy people or in people affected by pathologies that may or may not involve the motor system. However, there is no clear picture about the impact of CBD on these issues, even though it is frequently administered alone for medical uses and is sold with THC as a drug for pain caused by muscle spasms in multiple sclerosis [35], and was recently licensed as a drug for severe forms of infantile epilepsy refractory to treatment with other drugs [36]. In light of the opinion expressed above, we carried out a review describing the effects of CBD on locomotor activity with the aim to highlight the different effects of its use on motor activity. On this basis, we focused specifically on the effects of CBD on locomotor activity with the aim to improve the knowledge on this topic through information existing in the scientific literature. Findings describing the effects on locomotor activity of the CBD precursor cannabidiolic acid, and of CBD alone or in combination with THC, together with the effects of CBD on locomotor modifications induced by diseases and on locomotor changes induced by other substances, are discussed in the present article.

## 2. Materials and Methods

Bibliographic research was carried out independently by two researchers (blinded to the authors) using major scientific databases (PubMed, Scopus, and Google Scholar) and a search engine of peer-reviewed literature on life sciences and biomedical topics. The investigators used the keywords “motor activity”, “locomotor activity”, and “locomotion” in combination with “cannabidiol”. All articles written in the English language and published in peer-reviewed scientific journals describing effects of cannabidiol administration on motor activity published between January 2009 and December 2021 were collected and discussed.

## 3. Results

### 3.1. Effects of Cannabidiolic Acid, Precursor of Cannabidiol, on Locomotor Activity 

Cannabidiolic acid (CBDA), the acidic form and precursor of CBD, is a terpenophenolic compound synthesized by a CBDA synthase and represents the main phytocannabinoid in the fiber and hemp seed oil varieties [37]. Anticipatory nausea (AN) is a side effect experienced with chemotherapy; it can be triggered by smells, sights, and sounds linked to treatment. Pharmacotherapy currently used for AN is limited to benzodiazepines but does not produce satisfactory results; furthermore, it can be potentially responsible for the development of dependence [38]. With the aim to study the possible use of CBD instead of benzodiazepines in patients affected by AN, the effects of CBDA were studied in a preclinical AN model in which CBDA was administered orally in the dosage range of 0.05–5 mg/kg to adult male rats, while the 5-HT1A receptor antagonist WAY-100,635 or a saline vehicle were administered intraperitoneally (i.p.). CBDA had no effect on locomotor activity at any dose when assessed in an open field (OF) test, with no significant overall effect of dose observed for the number of lines crossed. CBDA also did not show any effect on motor co-ordination. However, a significant attenuation of anxiety-like behavior induced by WAY-100,635 was evident, with total time spent in the central sector increasing with a higher dose of CBDA. The results also showed that CBDA was well tolerated and did not cause benzodiazepine-like sedative effects, thus indicating its possible utilization in AN therapy [39].

In another study, the effects of acute and repeated intraperitoneal administration of CBDA (0.001–1 mg/kg) were investigated on spontaneous locomotor activity in mice. Locomotor activity was evaluated in OF boxes (24 × 24 × 24 cm) provided with 14 axes (Y and X) in a low-luminosity room. Horizontal (deambulations) and vertical (rearings) movements were automatically recorded for 45 min. The results showed no significant effect of CBDA on deambulations and rearings. On this basis, the authors concluded that CBDA treatment does not affect spontaneous locomotor activity [40]. In light of these studies conducted on laboratory animals, it is possible to assert that the precursor of CBD does not seem to have any effect on locomotor activity. Thus, it can be concluded that peripherally injected CBDA, the precursor of CBD, has no influence on spontaneous locomotor activity at doses ranging between 0.001 and 5 mg/kg of body weight. 

### 3.2. Effects of Cannabidiol on Locomotor Activity

In a recent study of the behavioral profile of CBD (15, 30, and 60 mg/kg; i.p.) as a potential drug of abuse evaluated in C57BL/6J mice, it failed to induce conditioned place preference (CPP), a form of conditioning test used to evaluate motivational effects. In the same group of experiments, spontaneous withdrawal symptoms and motor activity in the OF were also examined 12 h after the last i.p. CBD administration (30 mg/kg/12 h, for 6 days). Any withdrawal symptom was detected as well as any potential motor activity alterations. Data obtained from these experiments indicate that CBD does not have the characteristics of a drug of abuse [41]. These findings indicate that CBD cannot be placed in the category of drugs of abuse, as this categorization is absolutely arbitrary.

The effects of long-term treatment for six weeks with an i.p. dose of CBD of 20 mg/kg on behavioral effects were studied by using a set of motor, anxiety, and memory tests in adult mice (3 months and 5 months of age). The results showed that CBD administration did not influence motor activity evaluated by accelerating rotarod tests, while it reduced locomotor activity in the OF [42]. This is in contrast to the findings of Viudez-Martínez et al. [41] that CBD does not alter motor behavior 12 h after its administration of 30 mg/kg for 6 days in OF tests. While CDB-treated mice showed altered locomotion, their motor performance in the rotarod test was intact. CBD showed no effects on spatial learning and long-term memory assessed in the Morris water maze (MWM) test or on memory in the novel object recognition test. The object recognition test (ORT), or novel object recognition test (NORT), is used in mice for testing different phases of learning and memory [43]. Furthermore, CBD did not influence anxiety behavior in the dark–light box (DLB) and OF test. DLB is used to predict anxiolytic-like or anxiogenic-like activity of substances in mice [44]. The anxiety state was changed in the current group in the elevated plus maze (EPM) test, another test employed to measure anxiety-like behavior in rodents [45], and CBD treatment impaired prepulse inhibition (PPI), a test to analyze sensorimotor gating. The PPI paradigm consists of the normal suppression of the startle reflex (startle), which occurs when an intense stimulus (pulse), capable of evoking the reflex itself, is preceded by a weak sensory event, or pre-stimulus (prepulse). This mechanism provides a quantitative measure of central processing by filtering out irrelevant stimuli [46]. The authors’ results demonstrated that prolonged CBD treatment has no negative effect on the behavior of adult mice. In summary, these findings show that long-term CBD treatment did not produce side effects regarding motor activity or memory and did not cause anxiety behavior [42]. Taken together, these experiments indicate that peripheral long-term treatment with CBD influences locomotor activity, producing hypolocomotion, while acute or short-term treatment does not have the same effect. 

### 3.3. Effects of Cannabidiol on Locomotor Modifications Induced by Experimental Models of Diseases

Based on the finding that treatment with CBD of patients affected by Parkinson’s disease (PD) reduced the L-DOPA-induced psychotic symptoms, thus improving quality of life, it has been evaluated if its i.p. administration (0.5 or 5 mg/kg) could attenuate subcutaneous (s.c.) (1 mg/kg) reserpine-induced motor and cognitive impairments in rats, using an experimental model to study motor impairments and cognitive deficits associated with PD. The results showed that CBD reduced the increase in catalepsy behavior and in oral movements, but not hypolocomotion, and improved memory deficit in the discriminative avoidance task caused by reserpine. This indicates that CBD could be useful to counteract cataleptic side effects or reduce disturbances such as tardive dyskinesia caused by drug therapy necessary for PD patients. Antioxidant and anti-inflammatory activities were evoked to justify CBD effects on the reserpine model [47]. Another explanation of the protective effects of CBD in these experiments implies the possibility that the cannabinoid compound could upregulate mRNA levels for the antioxidant enzyme, superoxide dismutase [48]. 

With the aim to study the involvement of the serotonergic system, particularly 5-HT1A receptors, in the effects of CBD, this compound was given i.p. at the dose of 5 mg/kg for four weeks, and cognition and locomotion were studied in a model of hepatic encephalopathy induced by bile duct ligation (BDL) in mice. The results showed that CBD reduced impairment of cognition and locomotion caused by BDL and restored hippocampal expression of the tumor necrosis factor (TNF)-α receptor 1 and the brain-derived neurotrophic factor (BDNF) genes, which were increased and decreased after BDL, respectively. Beneficial effects of CBD were not associated with reduced hippocampal 5-HT1A expression, but effects were abolished by s.c. administration of 5-HT1A receptor antagonist WAY-100635 (0.5 mg/kg), thus suggesting the involvement of this receptor in CBD effects, while BDNF did not seem to account for the cognitive improvement induced by CBD. Previous experiments have shown that BDL lowers the expression of A2A adenosine receptor and that effects of CBD on cognition, locomotion, and TNF-α receptor 1 expression can be blocked by ZM241385, an A2A adenosine receptor antagonist. The authors’ conclusions based on these results were that chronic treatment with CBD can produce, through activation of 5-HT1A receptors, improvements in cognitive and motor function when they are reduced by hepatic encephalopathy [49].

Preclinical and clinical evidence indicate that CBD has a role in the therapy of demyelinating diseases such as multiple sclerosis (MS), a pathology of the central nervous system (CNS) affecting millions of people worldwide. In these patients, motor activity is frequently reduced, with a negative impact on quality of life [50]. Since none of the current treatments are effective in reducing this symptom, many physicians and patients turn to therapy with cannabis derivatives [51]. It has been observed that, in MS patients, CBD did not induce catalepsy or modify physiological parameters such as heart rate, blood pressure, and body temperature, nor was psychomotor activity negatively affected. Furthermore, high CBD doses up to 1.500 mg per day and chronic use were well tolerated [52].

Some studies investigated the potential behavioral effects of CBD on pathologies having symptoms including, alterations of locomotor activity such as fragile X syndrome (FXS) or MS. FXS is a neurodevelopmental disorder that affects intellectual, social, and physical development due to a mutation of the *FMR1* gene, for which treatment options are still limited [53,54] and alternative therapies are needed. Signs of FXS include anxiety and motor hyperactivity [55]. A study investigated the effects of i.p. CBD (5 or 20 mg/kg body weight) administration in adult male hyperlocomotive and hyperexplorative *Fmr1* KO mice and wild-type mice, an experimental model used to investigate FXS. OF, EPM, spontaneous alternation, social preference, and passive avoidance tasks were used as behavioral tests. *Fmr1* KO and WT mice were treated with one dose of a vehicle or CBD 30 min before the behavioral tests. *Fmr1* KO mice exhibited a slow-going locomotive adaptation to the unfamiliar habitat in comparison to controls. However, the results showed that acute CBD reduced anxiety induced by EPM, but it had no impact on locomotion or anxiety-related parameters of *Fmr1mice* in the OF experiments [56]. In this study, CBD administration resulted in a further reduction in anxiety-like behavior in both *Fmr1*-deficient and WT mice, without any effect on locomotor activity, social, or cognitive performance. These data suggest that CBD may have anxiolytic effects not dependent on *Fmr1*, and thus may be considered for use in individuals affected by FXS. 

Cannabinoids are known to produce dose-dependent effects, and the therapeutic potential of medium-dose CBD for Alzheimer’s disease (AD) transgenic mice has not yet been assessed in great detail. The effects of i.p. CBD (5 mg/kg) or a vehicle were investigated in 12-month-old APPSwe/PS1ΔE9 (APP/PS1) transgenic female mice and their control, commencing the treatment three weeks prior to the assessment of behavior consisting of sectors including anxiety, exploration, locomotion, motor functions, cognition, and sensorimotor gating. APP/PS1 mice showed increased β-amyloid formation accompanied by behavioral modifications [57]. APP/PSI mice displayed hyperlocomotion and anxiety-related behavior. In the pole test, CBD administration did not produce any significant effect on latency to inversion, time to descend, and latency to platform. In the accelerod, the performance of APPxPS1 mice increased in time compared to controls, but this, as well as impairments in sensorimotor gating of APPxPS1 mice, were not modified by CBD. Sensorimotor gating is the mechanism for filtering relevant from irrelevant information and is deficient in several psychiatric diseases [58]. Briefly, principal results were represented by the reversal of insufficient object recognition showed by APP/PS1 transgenic female mice obtained through chronic treatment with CBD 5 mg/kg. This indicates a potential beneficial effect in AD [59]. 

CBD’s anti-inflammatory activity suggests its possible role in the treatment of depression. To verify this hypothesis, 30 mg/kg CBD was given peripherally to mice 30 min before the administration of lipopolysaccharide (LPS; 0.83 mg/kg) used as a neuroinflammatory model. Successively, the effects on behavioral tests for depressive-, anhedonic-, and anxious-like behavior were studied together with an analysis of levels of several mediators such as NF-ĸB, IκBα, and PPARγ in nuclear and cytosolic fractions of cortical samples. Other mediators investigated were interleukin (IL)-6 and TNF-α levels in plasma and the prefrontal cortex, respectively. The precursor tryptophan (TRP) and its metabolites kynurenine (KYN) and serotonin (5-HT) were measured in the hippocampus and cortex by HPLC. The kynurenine (KYN)/tryptophan (TRP) ratio was used to evaluate indoleamine 2,3-dioxygenase activity and KYN/serotonin (5-HT) to measure the balance of both metabolic pathways. The results showed an increase in both KYN/TRP and KYN/5-HT ratios induced by LPS, thus indicating the enhancement of IDO activity. CBD reduced the immobility time in the tail suspension test and increased sucrose preference in the LPS model without affecting locomotion and central activity in the OF test. CBD diminished cortical NF-ĸB activation, IL-6 levels in plasma and the brain, and increased the KYN/TRP and KYN/5-HT ratios in the hippocampus and cortex in the LPS model. The results obtained from the above experiments show that CBD is able to produce antidepressant-like effects in the LPS neuroinflammatory model, associated with a reduction in the kynurenine pathway activation, IL-6 levels, and NF-ĸB activation, but without any influence on motor activity. In the OF, CBD treatment did not modify changes induced by LPS in the time spent in the center or distance traveled [60]. In Table 1 are summarized experiments conducted to study the effects of CBD on locomotor alterations induced by experimental models reproducing diseases. The results of CBD investigation in experimental models reproducing diseases offer interesting stimuli. Among these is the fact that chronic treatment with CBD can produce, via 5-HT1A receptors, improvements in cognitive and motor dysfunction caused by hepatic encephalopathy [49]. Another noteworthy aspect is represented by the production of anxiolytic effects in the absence of any influence on locomotor activity or social and cognitive performance and the ability to produce antidepressant effects without modifications to locomotor activity. Experiments carried out to investigate CBD effects on motor activity alterations induced by diseases deserve to be studied in further depth.

### 3.4. Effects of Cannabidiol Combination with Δ9-Tetrahydrocannabinol on Locomotor Activity

A study was conducted to investigate CBD’s potential effects on the reduction in telemetered motor activity and hypothermia induced by i.p. acute THC (10–30 mg/kg) administration in rats. CBD was administered contemporaneously with THC or with CBD/THC ratios of 1:1 or 3:1, or 30 min prior to THC. The results showed that CBD did not reduce hypothermia and hypolocomotion caused by THC. Motor activity was significantly reduced from the first hour after treatment with CBD/THC for 2–4 h after injection, as well as 3–4 h after THC only. In contrast to other findings, these experiments found no proof that CBD improves hypothermic and hypolocomotive THC effects. However, we cannot exclude that other time intervals or larger doses of CBD might have different results. Overall, there was no evidence from this study that an elevated CBD content in cannabis would provide protection from the physiological effects of THC [61]. 

Sex differences were investigated in THC/CBD interactions potentially influencing nociception measured through tail withdrawal and paw pressure tests and locomotor activity in rats. Two series of experiments were carried out: in the first series of experiments, i.p. CBD (0, 10, or 30 mg/kg) was given 15 min before i.p. THC (0, 1.8, 3.2, 5.6, or 10 mg/kg), and rats were tested for antinociception and locomotor activity 15–360 min post-THC injection. In the second series of experiments, i.p. CBD (30 mg/kg) was administered 13 h or 15 min before i.p. THC (1.8 mg/kg). Animals were subjected to evaluation for nociception and locomotor activity 30–480 min after THC administration. The results showed that THC-induced time– and dose-dependent analgesia was greater in female than male rats, agreeing with previous findings [62]. In the first experiment, in which multiple doses of CBD/THC association were given, CBD was shown to increase analgesia and hypolocomotion induced by THC with no sex differences. In the second series of experiments, in which a single dose of CBD/THC association was investigated using two different CBD pre-treatment times, CBD did not significantly modify THC-induced analgesia and hypolocomotion. Further experiments showed that CBD significantly modulated THC metabolism in both sexes. On the basis of these results, the authors suggested that CBD may enhance, in a sex-independent way, THC antinociceptive and hypolocomotive effects prolonging the duration of THC action [63].

Potential sex differences in response to cannabinoids have been investigated. With the aim to mimic human smoke inhalation, an electronic cigarette-based model in rats was used to evaluate thermoregulatory and locomotor responses to inhaled THC, CBD, and the association of the two compounds. Using radiotelemetry devices for the assessment of body temperature and locomotor activity, male and female rats were exposed to THC (12.5–200 mg/mL) or CBD (100, 400 mg/mL) vapor using a propylene glycol vehicle, varying the inhalation duration (10–40 min). Antinociceptive effects were measured using a tail withdrawal test following vapor inhalation. Blood samples withdrawn after inhalation were analyzed for THC content. The results showed that THC inhalation reduced body temperature and increased tail withdrawal latency in a dose-dependent way in both sexes in the same manner. When inhaled alone, CBD produced modest hypothermia and suppressed locomotor activity in both males and females. The combination of THC and CBD (ratio 1:4) significantly reduced temperature and locomotor activity in an additive way and to a similar extent in the two sexes. Plasma THC concentration did not differ across rat sexes. On the basis of the data obtained from the above-described experiments, the authors maintain that inhalation of THC or CBD, alone and in association, induces equivalent effects in male and female rats [64].

The potential development of a tolerance to THC behavioral effects and the putative CBD influence on THC effects has been studied in three groups of adolescent squirrel monkeys (*Saimiri boliviensis*). They were administered daily for 4 months with an intramuscular vehicle, THC (1 mg/kg), or THC + CBD (1 mg/kg + 3 mg/kg) to observe the effects of a daily high THC dose on performance in tasks of cognition (repeated acquisition, discrimination reversal), spontaneous behavior, and day/night activity. Activity was monitored day and night through a wireless activity tracker placed in a jacket. Motor activity was measured combining locomotion and environmental manipulation. The results showed that THC initially impaired the performance of adolescent monkeys in a cognitive test, but not their performance in a task of cognitive flexibility. THC reduced motor activity and increased sedentary behavior. Tolerance to these latter behaviors appeared weeks after the beginning of the daily administration. Co-administration of CBD did not influence THC effects on cognitive performance, motor activity, or tolerance, but abolished emesis caused by THC on the first day of daily treatment. The authors did not confirm antagonistic or potentiating effects of CBD on high doses of THC, except for the attenuation of THC-induced emesis [65].

Locomotor activity was investigated in adolescent (PND28) and adult (PND70) B6 mice after acute i.p. treatment with a vehicle, 10 mg/kg THC, 20 mg/kg CBD, or THC/CBD (10 mg/kg + 20 mg/kg; 1:2), with the aim to study CBD effects as well as THC/CBD associations, because the 1:2 THC to CBD ratio is present in commercially available products for recreational and medical use [66] in some countries. Effects of CBD, THC, and THC/CBD were studied in the OF after 30 min of drug pre-treatment. Acute CBD alone and in combination with THC resulted in robust effects on locomotor behavior. The results showed that THC and CBD significantly reduced total locomotion in both age groups. In all groups, with the exception of adults administered THC/CBD, total locomotion was significantly positively correlated with percent time in the center of the OF (*p* < 0.01). THC and THC/CBD significantly decreased the percentage of total locomotion in the center of the OF in adults, indicating anxiogenic effects. Regarding the total locomotion in the OF, for CBD, a significant interaction [*F*(3,61) = 3.42, *p* < 0.05] and main effect of age [*F*(1,61) = 9.20, *p* < 0.01] were observed, with more movement in adult mice, together with evidence that 5 mg/kg dose reduced total locomotion in adults [*t*(31) = 2.67, *p* < 0.05, *d* = 1.31] [67]. The results of this study do not clarify the effects of CBD on motor activity because they are not discriminative with respect to the effects of the association of THC/CBD. Moreover, these findings show that THC/CBD would lead to increased impairment, in conflict with the hypothesis that a THC/CBD combination would result in reduced impairment. In another study, it was shown that simultaneous i.p. CBD administration (10 mg/kg) enhanced hypolocomotion caused by i.p. THC (10 mg/kg) in rats and at the same time reduces other psychopharmacological THC activity such as anxiogenic effects [68]. In summary, laboratory experiments show that concomitant CBD/THC administration does not influence, in a significant way, hypothermic THC effects, as well as its effects in reducing locomotor activity. The effects of THC/CBD association on locomotor activity are summarized in Table 2.

### 3.5. Effects of Cannabidiol on Locomotor Changes Induced by Other Substances

The effects of various i.p. doses of CBD on motor activity have been compared with those of the selective 5-HT1A receptor agonist, 8-hydroxy-2-(di-n-propylamino) tetralin (8-OH-DPAT), i.p. injected in rats. In the same study, the researchers also investigated if the potential CBD effects on motor activity could be sensitive to 5-HT1A receptor blockade in comparison with CB1 receptor antagonism, and whether CBD was able to enhance the effect of a sub-effective dose of 8-OH-DPAT (1 mg/kg). The results showed that only high doses of i.p. CBD (>10 mg/kg) changed motor behavior, which was measured through a computer-aided actimeter with alterations restricted to vertical activity (rearings), and only modest changes occurred in other parameters. The compound 8-OH-DPAT caused comparable effects but were limited to vertical activity, with no influence on other motor aspects, and were always more potent than CBD. The effects of 8-OH-DPAT and CBD on vertical activity were restored by i.p. WAY-100,635 (0.5 mg/kg), 5-HT1A receptor antagonist, but CBD effects on vertical activity were not brought back by the CB1 receptor antagonist rimonabant (0.1 mg/kg; i.p.). In these experiments, the effects of 8-OH-DPAT on vertical activity were accompanied by the enhancement of serotonin in the basal ganglia. This effect was not produced by CBD. Finally, the highest dose of CBD increased the effects on motor activity of a sub-effective dose of i.p. 8-OH-DPAT (0.1 mg/kg), thus causing changes in serotonergic transmission in the basal ganglia. The results of this study indicate that CBD may affect motor activity, particularly vertical activity, and that this effect could be linked to its activity on the 5-HT1A receptor [69] (Table 3). This mechanism of action has been evoked for CBD’s anti-emetic, antidepressant, and anxiolytic properties [70,71].

A large body of evidence suggests that CBD may have a role in the development of new pharmacological approaches for the therapy of psychiatric disorders [72]. In a preclinical study, different doses of CBD (3, 5, 10 mg/kg) were given orally to male adult rats concomitantly with oral haloperidol 5 mg/kg to assess their effects on OF, rotarod tests, EPM, and ORT behavioral tests. Oral doses of CBD 5 mg/kg or the antipsychotic drug haloperidol 5 mg/kg alone, not in combination, were also investigated for their effects on the same tests. All behavioral assessments were performed 24 h after treatments. The OF test was used to assess locomotion. The count of line crosses and frequency of rearing is generally calculated as a measure of locomotor activity, exploratory activity, and state of anxiety. Detection of a high frequency of these behaviors indicates increased locomotion and exploration associated with a lower degree of anxiety [73]. The rotarod test was also used for the assessment of forced locomotor activity in rodents [74] using a rotarod treadmill device, and time spent on the treadmill before falling was measured for each animal. The results of the OF test showed that animals treated with all drug treatments spent more time in the open arms compared to rats of the control group treated with distilled water. Moreover, rats treated only with haloperidol spent significantly more time in the open arms in comparison with rats treated with the combination CBD/haloperidol or the group treated with CBD alone. There was also a statistically significant difference in the mean time spent in closed arms between the two kinds of treatments. Data from rotarod tests showed that rats treated only with haloperidol 5 mg/kg spent the shortest time on the treadmill of all the animals, thus indicating that the high dose of haloperidol reduced motor activity in rats. Treatment with the highest dose of CBD (10 mg/kg) in association with haloperidol (5 mg/kg) resulted in the most time spent on the treadmill, suggesting a reduction in haloperidol-induced hypolocomotion produced by CBD administration. Data from behavioral tests were accompanied by CBD reducing the effects of hyperglycemia induced by haloperidol. Furthermore, in the same group of experiments, the administration of CBD alone was associated with antioxidant activity because increased brain α, α-diphenyl-β-picrylhydrazyl (DPPH) free radical scavenging activity was detected [75]. These experiments indicate that CBD can improve motor deficits induced by haloperidol and suggest that this cannabinoid deserves to be investigated as a useful agent to protect from extrapyramidal side effects and long-term movement disorders induced by antipsychotic drugs.

In another study, the effects of CBD on motor activity, somatic signs, and anxiety-like behavior were evaluated 6 h after the last dose of progressively increased i.p. cocaine (15 to 60 mg/kg) administered daily for 12 days. Concomitantly, gene expression changes in dopamine transporter (DAT) and tyrosine hydroxylase (TH) in the ventral tegmental area and in CB1R and CB2R in the nucleus accumbens were evaluated. In this experimental model, mice exposed to cocaine withdrawal presented with increased motor activity, somatic withdrawal signs, and high anxiety-like behavior. Administration of CBD (10, 20, and 40 mg/kg; i.p.) normalized motor and somatic sign disturbances and produced anxiolytic effects. Furthermore, CBD abolished the increase in DAT and TH gene expression induced by cocaine withdrawal, regulated the reduction of CBR1, and caused upregulation of CBR2 gene expression analyzed by real-time PCR. For the authors, the results show that CBD reduces behavioral and gene expression alterations induced by cocaine withdrawal [76]. It is noteworthy that CBD restores normal motor activity reducing hyperlocomotion associated with anxiety induced by cocaine withdrawal. This finding could be linked to others’ suggestions that CBD reduces rewarding effects induced by cocaine through CB2, 5-HT1A, and TRPV1 receptor mechanisms [77].

CBD has also been investigated for its antipsychotic properties. Experiments were recently carried out to study the role of CBD on motor alterations induced by the psychotomimetic substance ketamine with the objective to clarify the role of CBD in ketamine motor effects and glutamatergic signaling in brain regions such as the prefrontal cortex, nucleus accumbens, and dorsal and ventral hippocampus. In this study, i.p. CBD alone decreased motor activity at the highest dose tested (30 mg/kg), while i.p. ketamine (30, 60 mg/kg) enhanced locomotor activity measured in an OF apparatus. Furthermore, findings from these experiments showed that CBD pre-treatment did not abolish hyperactivity caused by ketamine but prolonged it over time. The authors also showed that pre-treatment with CBD restored ketamine changes in NMDA, NR1, and NR2B receptor expression and ERK phosphorylation, thus suggesting that CBD is able to increase ketamine motor stimulation and return to normal ketamine effects on glutamatergic signaling and neuroplasticity-related markers [78]. These experiments suggest that potential antipsychotic CBD effects may be associated with its regulatory role in glutamatergic signaling in brain regions linked to motor output. In another study, S-ketamine (2.5, 3, 5, 10, 30 mg/kg) and cannabidiol (3, 10, 30 mg/kg) were injected alone or in combination into mice. Animals were treated with the AMPA receptor antagonist, NBQX, five minutes before CBD or S-ketamine injection. CBD produced significant dose-dependent antidepressant effects measured through forced swimming but did not show any influence of motor activity quantified with the OF test, while a higher dose of S-ketamine showed antidepressant effects accompanied by increased motor activity. NBQX antagonizes the antidepressant effects of both substances. Interestingly, previous treatment with CBD (10 mg/kg) reduced ketamine-induced increases in motor activity but preserved ketamine-induced antidepressant effects [79]. These results suggest that AMPA signaling is, as happens for ketamine, responsible for CBD antidepressant activity and that on the basis of prevention of hyperactivity induced by ketamine, CBD could be explored as a potential adjuvant for mood disorders. The effects of CBD on locomotor alterations induced by substances other than THC are summarized in Table 3.

**Table 3:** Effects of cannabidiol (CBD) on locomotor alterations induced by other substances.

Species	Substance	CBD	Locomotor Activity	Reference
Rat	Single i.p. rimonabant (0.1 mg/kg; i.p.)	Single i.p. CBD (20 mg/kg)	No effect of rimonabant on CBD-induced reduction of vertical activity.	[69]
	Single i.p. WAY-100,635 (0.5 mg/kg)	Single i.p. CBD (20 mg/kg)	WAY-100,635 restored CBD-induced reduction of vertical activity.	
	Single i.p. 8-OH-DPAT (1 mg/kg)	Single i.p. CBD (20 mg/kg)	CBD potentiated 8-OH-DPAT reduction of vertical activity.	
Rat	Single oral haloperidol (5 mg/kg)	Single oral CBD (3, 5, 10 mg/kg)	Haloperidol or CBD caused an increase in time spent in the open arms. This effect was slightly reduced by concomitant CBD/haloperidol administration.	[75]
			Haloperidol reduced motor activity. CBD (5 mg/kg) antagonized hypolocomotion induced by haloperidol.	
Mouse	Daily progressive i.p. dose of cocaine (15 to 60 mg/kg) three times a day for 12 days	Single i.p. CBD (10, 20, and 40 mg/kg) given 270 min after the last cocaine administration	All doses of CBD blocked the increase in total distance values induced by cocaine withdrawal.	[76]
Rat	Single i.p. ketamine (30, 60 mg/kg) alone or 10 min after CBD	Single i.p. CBD (10 or 30 mg/kg) alone or 10 min before ketamine	CBD (30 mg/kg) alone decreased motor activity; ketamine (30, 60 mg/kg) alone increased motor activity. CBD did not reverse ketamine-induced short-lasting hyperactivity but prolonged it over time.	[78]
Mouse	Single i.p. S-ketamine (2.5, 3, 5, 10, 30 mg/kg)	I.p. CBD (3, 10, 30 mg/kg) 30 min before S-ketamine	CBD produced antidepressant effects without any influence on motor activity. Higher dose of S-ketamine showed antidepressant effects with increase in motor activity.	[79]

i.p. = intraperitoneal; i.m. = intramuscular.

## 4. Discussion

There are a growing number of products based on CBD, such as food, cosmetics, and drinks, and they are consumed daily by the population [80]. This circumstance highlights the need for studies that can increase the knowledge of potential effects on various outcomes, including the influence of CBD on motor activity. This kind of information is particularly important to prevent possible motor disturbances in people affected by diseases that include motor alterations in their symptoms and in people planning regular motor activity as amateurs or as athletes.

In this article, CBD’s effects on motor activity were analyzed, separating those of CBD or its precursor CBDA from effects on diseases with motor alterations and effects of CBD concomitantly used with THC or other substances. Experimental data on laboratory animals show that the precursor CBDA has no influence on spontaneous locomotor activity at doses ranging between 0.001 and 5 mg/kg of body weight. Short-term and long-term (6 weeks) CBD administration in laboratory animals does not seem to influence spontaneous motor activity except when the CBD is inhaled [39,40], thus indicating that CBD effects can change according to the different mechanism of administration. Another interesting aspect is the finding that chronic treatment with CBD is able to produce, via 5-HT1A receptors, improvements in motor as well as cognitive dysfunction in an experimental model of hepatic encephalopathy.

The findings reviewed in this article suggest that CBD may have anxiolytic effects not dependent on the *Fmr1* gene, which is mutated in FXS, characterized by signs such as anxiety and motor hyperactivity [56]. In other experiments, when administered to animals mutant for Scn1a^+/−^, a common epilepsy gene, CBD improved social behavior and reduced hyperlocomotion [81].

The preclinical studies discussed in this article demonstrate that CBD administration is able to reduce catalepsy behavior and oral movements induced by reserpine [47]. These results suggest CBD’s potential utility in counteracting motor disturbances induced by pharmacological therapy in PD patients. These beneficial effects are corroborated by evidence that CBD changes the conductance of voltage-dependent anion channel 1, involved in the pathophysiology of movement disorders [82]. CBD is also implicated in the activation and upregulation of PPARγ, a receptor known to be associated with the appearance of oral dyskinesia [75]. Moreover, CBD also showed potential antidepressant effects without any changes in locomotor activity [79], thus suggesting the possibility of using CBD in these diseases without concern for motor activity. Furthermore, CBD was also shown to produce anti-anxiety effects without a negative influence on motor activity [60]. Results from laboratory studies investigating the potential influence of CBD on THC’s effects on motor activity show that high doses of CBD can potentiate hypolocomotion induced by THC. A reduction in vertical activity has been revealed in rodents treated with peripheral CBD. Observation of rearing-up vertical activity is considered a useful method to measure anxiety states in mice [83]. This effect also appears to be linked to a mechanism involving the serotonergic system because a CBD-induced reduction in vertical activity can be restored by the co-administration of 5-HT1A receptor antagonist WAY-100,635. Experiments also indicate that CBD could increase hyperactivity over time caused by ketamine administration. Finally, it is noteworthy that CBD was found to produce antidepressant effects, probably mediated through the AMPA signaling pathway, without any influence on motor activity. CBD is usually considered as a substance having no effects on locomotor activity because of its poor affinity for CB1 or CB2 receptors despite its activity at the TRPV1 receptor. The evidence discussed in this paper indicates that this phytocannabinoid probably interacts with the serotonin 5-HT1A receptor, causing part of its positive effects and influencing motor activity through the role of serotonergic signals in the basal ganglia. Moreover, the effects of CBD prolonging increases in locomotion induced by ketamine, associated with potential antischizophrenic properties, suggest its possible influence on glutamatergic system involvement in motivation and motor activity [78]. CBD has a high affinity for the GPR55 receptor [84], and expression of this receptor was found to be profoundly downregulated in the striatum of rodents in experimental PD models, raising the hypothesis that GPR55 might be involved in the development of this pathology and that CBD could be a potential therapeutic tool for PD [85].

Finally, clinical studies on patients resistant to drug treatment for epilepsy showed that the effects of CBD can be affected by pharmacogenetic variations influencing the expression of CBD targets [86]. This variability can produce either a lack of efficacy or the occurrence of adverse effects. In this regard, preclinical studies have suggested that patients with mutations in SCN8A, the gene encoding for voltage-gated sodium channel subunit 8 and that is associated with seizures, movement disorders, and autism, could result in a reduction in seizures with CBD treatment [87]. This change is also accompanied by an improvement in social behavior and a reduction in hyperactivity. From this point of view, the potential use of CBD needs further research in terms of pharmacogenetic aspects associated with its effects on motor activity.

## 5. Conclusions

In conclusion, CBD does not seem to have a significant influence on motor activity when administered or consumed alone. There is evidence of positive effects of long-term CBD treatment on brain- and nervous system-associated diseases such as multiple sclerosis (with THC), brain ischemia, and epilepsy. Moreover, preclinical findings suggest that CBD could be further investigated for its effects in counteracting hypomobility associated with neuropathologies such as encephalopathy or Parkinson’s disease.

## Figures and Tables

**Table 1:** Effects of cannabidiol (CBD) on locomotor alterations induced by experimental models reproducing diseases.

Species	Experimental Model or Disease	CBD Administration	Locomotor Activity	Reference
Rat	Hypolocomotion associated with Parkinson’s disease induced by s.c. reserpine 1 mg/kg	Single i.p. 0.5 or 5 mg/kg or vehicle	Reduction of catalepsy and tardive dyskinesia	[47]
Mouse	Hepatic encephalopathy induced by bile-duct ligation	Daily i.p. 5 mg/kg or vehicle for four weeks	Reduction of hypolocomotion	[49]
Fmr1 knock out mouse	Fragile X syndrome	Single i.p. 5 or 20 mg/kg or vehicle	No effect on hyperlocomotion	[56]
APP/PS1 Mouse	Alzheimer disease	Daily i.p. 5 mg/kg or vehicle for 3 weeks before the beginning of experiments and during behavioral test assessment	No effect on locomotor activity	[59]
Mouse	Depressive and anxious-like behavior induced by LPS	Single i.p. 30 mg/kg or vehicle 30 min before LPS	No effect on changes in time spent in the center or distance travelled induced by LPS	[60]

i.p. = intraperitoneal; s.c. = subcutaneous; LPS = lipopolysaccharide.

**Table 2:** Effects of Δ9-tetrahydrocannabinol/cannabidiol (THC/CBD) combination on locomotor activity.

Species	THC	CBD	Locomotor Activity	Reference
Rat	Single i.p. 10–30 mg/kg	Simultaneous single i.p. CBD 10–30 mg/kg with THC; i.p. CBD 10–30 mg/kg or 30–90 mg/kg, 30 min prior to THC	No effect on hypolocomotion induced by THC	[61]
Mouse	Single i.p. THC (0, 1.8, 3.2, 5.6 or 10 mg/kg)	Single i.p. CBD (30 mg/kg) 15 min before THC	CBD enhanced hypolocomotion induced by low to moderate THC doses	[63]
	Single i.p. THC 1.8 mg/kg	Single i.p. CBD (0, 10 or 30 mg/kg) 13 h or 15 min before THC	CBD did not change significantly THC effects on locomotor activity.	
Rat	Vaporized THC (12.5, 25, 50, 100, 200 mg/mL) 0.125 mL in a 40 min session	CBD (100, 400 mg/mL) 0.125 mL in a 40 min session	CBD alone when inhaled suppressed locomotor activity. THC and THC/CBD (1:4) inhalation decreased total locomotion in the center of the OF.	[64]
Monkey	Increasing i.m. THC dose (0.1–1 mg/kg for four months	Increasing i.m. CBD dose (0.3–3 mg/kg) for four months	Tolerance to hypolocomotion induced by THC appeared after weeks. CBD did not influence THC hypolocomotion or occurrence of tolerance.	[65]
Mouse	Single i.p. THC (10 mg/kg)	Simultaneous i.p. CBD (20 mg/kg)	CBD did not modify hypolocomotion induced by THC.	[67]
Mouse	Single i.p. THC (10 mg/kg)	Simultaneous single i.p. CBD (10 mg/kg)	CBD enhanced hypolocomotion induced by THC.	[68]

i.p. = intraperitoneal; i.m. = intramuscular.
